# Non-viral systems for intracellular delivery of genome editing tools

**DOI:** 10.18699/vjgb-24-28

**Published:** 2024-04

**Authors:** I.H. Shaikhutdinov, P.V. Ilyasov, O.V. Gribkova, L.V. Limareva

**Affiliations:** Samara State Medical University of the Ministry of Healthcare of the Russian Federation, Samara, Russia; Samara State Medical University of the Ministry of Healthcare of the Russian Federation, Samara, Russia; Samara State Medical University of the Ministry of Healthcare of the Russian Federation, Samara, Russia; Samara State Medical University of the Ministry of Healthcare of the Russian Federation, Samara, Russia

**Keywords:** metal-organic frameworks, vesicles, nanoparticles, viral vectors, gene editing, металл-органические каркасные полимеры, везикулы, наночастицы, вирусные векторы, редактирование генов

## Abstract

A hallmark of the last decades is an extensive development of genome editing systems and technologies propelling genetic engineering to the next level. Specific and efficient delivery of genome editing tools to target cells is one of the key elements of such technologies. Conventional vectors are not always suitable for this purpose due to a limited cargo volume, risks related to cancer and immune reactions, toxicity, a need for high-purity viral material and quality control, as well as a possibility of integration of the virus into the host genome leading to overexpression of the vector components and safety problems. Therefore, the search for novel approaches to delivering proteins and nucleic acids into cells is a relevant priority. This work reviews abiotic vectors and systems for delivering genome editing tools into target cells, including liposomes and solid lipid particles, other membrane-based vesicles, cell-penetrating peptides, micelles, dendrimers, carbon nanotubes, inorganic, polymer, metal and other nanoparticles. It considers advantages, drawbacks and preferred applications of such systems as well as suitability thereof for the delivery of genome editing systems. A particular emphasis is placed on metal-organic frameworks (MOFs) and their potential in the targeted intracellular delivery of proteins and polynucleotides. It has been concluded that further development of MOF-based vectors and technologies, as well as combining MOFs with other carriers can result in safe and efficient delivery systems, which would be able to circulate in the body for a long time while recognizing target cells and ensuring cell-specific delivery and release of intact cargoes and, thereby, improving the genome editing outcome.

## Abstract (ru)

Последние десятилетия отмечены интенсивным развитием технологий и систем редактирования генов, которое вывело генную инженерию на новый уровень. Важным звеном этих технологий является специ- фичная и эффективная доставка компонентов таких систем в клетки-мишени. Традиционные векторы не всегда подходят для этой цели ввиду ограниченного объема полезной нагрузки, рисков, связанных с канцерогенезом и иммуногенностью, токсичности, необходимости высокой степени очистки и оценки качества полученных вирусных носителей, а также возможности встраивания вируса в геном хозяина, что может приводить к сверх- экспрессии компонентов вируса и проблемам с безопасностью. Это обусловливает актуальность поиска новых средств внутриклеточной доставки белков и нуклеиновых кислот. В данной работе приведен обзор абиоти- ческих векторов и систем доставки инструментов для редактирования генома, включая липосомы и твердые липидные наночастицы, мембранные везикулы иной природы, пептиды, проникающие в клетки, мицеллы, ден- дримеры, углеродные нанотрубки, неорганические, полимерные и другие наночастицы, металл-органические каркасные полимеры. Рассмотрены их преимущества, недостатки и предпочтительные области применения, а также возможность их использования для доставки систем редактирования генов. Особое внимание уделено металл-органическим каркасным полимерам и их потенциалу в качестве средств избирательной внутрикле- точной доставки белков и полинуклеотидов. Сделан вывод о том, что дальнейшее развитие таких векторов и технологий на их основе может привести к появлению безопасных и эффективных систем доставки, способных длительно циркулировать в крови и распознавать клетки-мишени, обеспечивая адресное высвобождение по- лезной нагрузки в неизменном состоянии и тем самым улучшая результаты редактирования генов.

## Introduction

The last decades were marked by the development of novel strategies and genome editing tools for treatment of hereditary and acquired diseases. Such tools include but are not limited to specific synthetic oligonucleotides, recombinant zinc finger nucleases (ZFNs), transcription activator-like effector nucleases (TALENs), genome editing systems based on clustered regularly interspaced short palindromic repeats and associated enzymes (CRISPR/Cas), and genomic DNA base editors. Their efficacy strongly depends on the methods for delivery thereof into the target cells and tissues. Currently, various approaches and vector systems, having their specific advantages and drawbacks, are being used for these purposes.

The major challenges of such delivery inherent for genome editing tools include a large size of CRISPR/Cas or TALEN components, a large negative charge of RNAs, immunogenic potential, low efficacy, and off-target side effects (Singh D. et al., 2016). Transfection of such tools is also complicated by multiple factors impeding the cell and nucleus penetration by nucleic acids and proteins, with additional issues and limitations often conferred by the delivery methods and systems which were supposedly designed to facilitate the passing of the barriers. All these reduce the efficacy of genetic manipulations with the target cells

The above tools have been delivered into the cells using a variety of techniques including electroporation, mechanoporation, microinjection, hydrodynamic injection, sonoporation, etc. (Moscoso, Steer, 2020). Among them, the most common ones are electroporation, due to its ease of use, high efficiency of in vivo transfection and genome editing, and microinjection, which allows to inject DNA directly to the nucleus. Particularly, when using CRISPR/Cas, microinjection allows to control the amount of Cas/sgRNA complex to be injected and to overcome the molecular weight limitations (Wang H.X. et al., 2017). The main limitations of the electroporation are low cell viability after the manipulations and a need to adjust the technique to the particular cells and vectors. In the case of microinjection, the limitations include relatively high complexity, labor intensity and cost of the procedure. Moreover, these methods are not suitable for all tissues of the body in vivo, and they have generally been used for small animal genome editing.

Another approach to the delivery of nucleic acids and proteins into the cells is based on vectors, which are able to penetrate the cells without using any ancillary tools. Conventionally, this assumes the use of viral vectors as they have an evolutionarily optimized machinery for introducing their genetic material into host cells. They are highly stable, can readily penetrate biological barriers, drive efficient transfection and induce long-term gene expression, and are able to infect both proliferating and nonproliferating cells (Huang et al., 2011). At the same time, serious disadvantages of the viral vectors include restricted cargo volume, cancer risk, immunogenic properties, toxicity, and a need for high purification and quality control of the vector used. Moreover, many viral vectors integrate themselves into the target cell genome, which may result in the overexpression of the genome editing system components and potentially cause safety issues (Hanlon et al., 2019).

Therefore, search for and development of alternative nonviral vector systems that would be able to bind nucleic acids and proteins and release them in a controlled manner is a relevant priority. Such delivery systems should have a number of advantages, particularly, an ability to load and deliver large molecules, an ease of preparation, low toxicity, minimal immune reactivity, and a possibility of customization of the properties defining their practical implementation. Almost all abiotic vectors have a positive charge required for electrostatic DNA complexing (Mintzer, Simanek, 2009). In contrast with other delivery systems, they are able to transfer the editing complexes in various forms including DNA, ribonucleoproteins and mRNA (Liu C. et al., 2019; Niggemann et al., 2020). Notably, non-viral vectors perform transient delivery, which is preferred in some cases of genome editing. Genome editing components are degraded shortly after cell penetration, thereby reducing the off-target effects (Mout et al., 2017a). In addition, many non-viral vectors can be commercially manufactured with the defined parameters

Genome editing tool delivery systems such as liposomes and solid lipid particles, other membrane-based vesicles, cellpenetrating peptides, micelles, dendrimers, carbon nanotubes, inorganic, polymer, metal and other nanoparticles, and metalorganic frameworks (MOFs) are especially noteworthy. The Table shows advantages and drawbacks for some of them, with more details provided in the following sections.

**Table 1.**
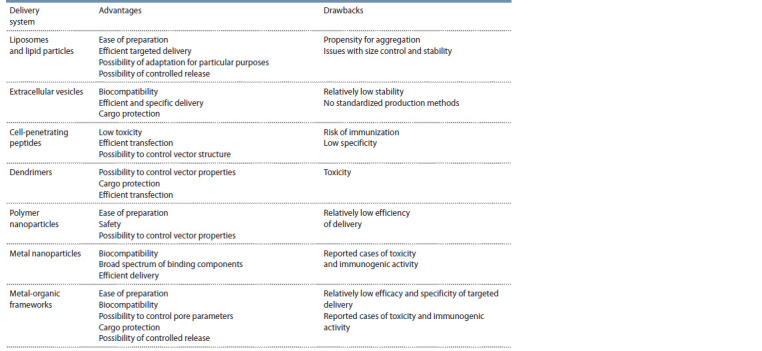
Summary of advantages and drawbacks of non-viral systems for delivery of genome editing tools

## Lipid-based nanoparticles

Liposome-mediated gene transfer was one of the first strategies for introducing foreign genetic material into target cells (Mintzer et al., 2009). Currently, composition of the liposomes used for this purpose widely varies and may include, e. g., cationic lipids, polyethylene glycol, cholesterol, phospholipids, dioleylphosphatide acid, etc. (Kim et al., 2020; Patel et al., 2020). They readily penetrate target cells and ensure specific delivery, which significantly reduces effects in off-target tissues and organs vs. DNA vector-based delivery of CRISPR (Yeh et al., 2018). This supports the significance of studies of lipid carriers as the delivery systems for genome editing tools. For example, in a study (Andey et al., 2019), a lipoplex was synthesized incorporating small interfering RNA (siRNA) of the SOX2 transcription factor for target therapy of SOX2- enriched lung tumors in CB-17 nude mice. 85 % of animals administered with the lipoplex showed a reduction in their tumor weight and volume, which was associated with the reduction in SOX2 protein expression. A review (Lu Z.R. et al., 2021) provides a summary on the use of DODAP and other ionizable pH-sensitive lipids, which can also respond to other changes in the environment to produce the nanoparticles incorporating siRNA molecules for cancer therapy and targeted oncogene silencing. The use of lipid nanoparticles for the delivery of regulatory RNAs into the cells is also described in (Wang C. et al., 2021; Eygeris et al., 2022), as well as in an integrated review devoted to the use of lipids and their derivatives for the RNA delivery (Zhang Y. et al., 2021). These works suggest different approaches and advantages of lipid nanoparticles, including the relative ease of the targeted delivery, possibility of controlled release, protection from aggregation and elimination from the blood stream by the host immune system due to the use of PEG and other protective molecules, endosomal escape, etc.

Commercially available lipid nanovehicles include, e. g., Lipofectamine 2000, Lipofectamine 3000, RNAiMAX, which are used to deliver CRISPR/Cas9 components as a mix of Cas9 mRNA, gRNA and ribonucleoproteins into various cells (Yu X. et al., 2016). Lipid nanoparticles allow simultaneous encapsulation and delivery of several RNA types (mRNA and siRNA) into the target cells (Ball et al., 2018). In addition, they can be adapted to a particular way of administration, cell type and genome editing tool (Liu J. et al., 2019; Lokugamage et al., 2021).

However, the issues with controlling the size, uniformity and stability of the lipid nanoparticles restrict their use, particularly for in vivo gene therapies. Sometimes, these issues may be addressed by modification of the nanoparticle surface with PEG and other polymers, or by using nanoparticle cores made of a different material (for example, gold or polystyrene) and forming lipid layers with the incorporated cargoes over the core (Yan et al., 2022); however, this generally hampers preparation and use of such carriers and, in a number of cases, it would be practical to omit such systems in favor of other biotic and abiotic vectors

## Extracellular vesicles: exosomes and microvesicles

A number of scientists propose natural cell membrane-derived vesicles, including exosomes, microvesicles and apoptotic bodies, as the carriers to deliver genome editing tools in vitro and in vivo while protecting them in the biological fluids and extracellular matrix.

Exosomes are extracellular vesicles produced by all cells. They were initially considered as drug carriers due to their small size, perfect biocompatibility, ability to transfer biomolecules into the cells and specific expression of the cell surface receptors. Further studies have shown that the exosomes carrying siRNAs can protect their cargo from enzymatic cleavage (half-life > 48 h), while naked siRNAs have half-lives of less than 6 h (Yang Z. et al., 2016). Moreover, encapsulation of siRNA into the exosomes improved its absorption by the cells

Kamerkar S. et al. have constructed exosomes carrying a siRNA that targeted proto-oncogenic KRAS GTPase. These exosomes inhibited tumor development in various mouse pancreatic cancer models and significantly increased overall survival (Kamerkar et al., 2017).

To reduce immunogenic potential of exosomes carrying siRNAs and proteins in mice with Alzheimer’s disease, mouse dendritic cell-derived vesicles were used (Alvarez-Erviti et al., 2011). In this case, the proteins characteristic for target cells were fused to Lamp2b, which is abundant in exosome membranes. Such modification resulted in efficient cell typespecific gene knockdown while minimizing host immune response.

Moreover, the studies have shown that modified exosomes can transfer guide RNA and Cas9 protein between HuH7 line cells (Chen R. et al., 2019). This work describes intercellular delivery of CRISPR/Cas9 components ensuring cleavage of hepatitis B virus and papilloma virus DNA in the infected cells.

Although the in vivo transfer of genome editing tools using exosomes showed no apparent side effects, the loading and targeting efficacy of such delivery systems is understudied. Another limitation of the clinical use of exosomes is the lack of standardized methods for their isolation and analysis (Doyle, Wang, 2019). Therefore, detailed studies of mechanisms and consequences of the vesicle-mediated delivery are needed as the result of such delivery may strongly depend on the cargo and cells used

Microvesicles, another type of extracellular vesicles, are also of interest as potential delivery means. In contrast to exosomes, which are derived from endosomes, microvesicles are formed directly from the plasma membrane. They are larger than exosomes, which allows increasing their actual payload (Kanada et al., 2015). The potential of epitheliumderived microvesicles as a delivery system for CRISPR/Cas9 and sorafenib was assessed in the hepatocellular carcinoma model (Samuel et al., 2020), which showed enhanced microvesicle homing towards the tumor cells and a synergy of the agents loaded. A number of works also describes the use of microvesicles for the delivery of siRNAs and miRNAs into the cells to regulate intracellular and tissue processes, such as fibrosis, tumor growth inhibition, and the like (Vader et al., 2017; Stolzenburg, Harris, 2018).

## Cell-penetrating peptides

An efficient delivery system must perform in a variety of tissues, ensuring rapid cargo release, be functional with low payload doses, non-toxic and easy to use in clinical practice. These properties, among others, are common for cell-penetrating peptides (CPPs). These peptides can bind to different molecules, interact with membrane structures, penetrate cells and deliver their cargo into the cytoplasm or nucleus. There are a lot of such peptides that can bind the molecules of interest in a covalent or non-covalent manner and translocate into the cells by means of direct membrane crossing, endocytosis, or formation of a transport channel in the membrane. Due to a number of their advantages, CPPs are widely used in studies to transfer small RNAs/DNAs, plasmids, antibodies, and nanoparticles into the cells. Their beneficial properties include controllable low toxicity, high transfection efficacy, and structural flexibility (Lopez-Vidal et al., 2021).

In a study (Ramakrishna et al., 2014), CPP was conjugated with a modified Cas9 protein and gRNA to induce gene disruptions in the target site in embryonic stem cells, dermal fibroblasts, HEK293T, HeLa, and human embryonic carcinoma cells. This genome editing tool delivery system efficiently changed target gene expression with the reduction in off-target mutation rate vs. the plasmid-based transfection

Lopez-Vidal E.M. et al. successfully used a conjugate of a short synthetic peptide with low arginine content and antisense oligonucleotides for the transfection of HeLa654 cells and cardiac tissue of transgenic mice in vivo (Lopez-Vidal et al., 2021).

The efficacy of CPPs as vectors for gene delivery was shown for their complexes with modified viruses, plasmid DNA, small interfering RNAs, oligolucleotides, DNA origami platforms, full-length genes, etc. (Taylor, Zahid, 2020). Features limiting their use include their high molecular weight, risk of host immunization, and insufficient delivery specificity.

## Dendrimers

Dendrimers are another example of abiotic vectors. They are generally characterized by advantageous safety, lack of immunogenic potential, high efficacy, reproducibility, controllable size, broad range of possible modifications, an ability to form stable complexes with different molecules and deliver several molecule types (e. g., drug and gene) at once (Abedi-Gaballu et al., 2018). They can also promote release from endosomes after cell penetration due to the proton sponge effect. Dendrimer molecules can associate with different moieties and ligands, including antibodies, signaling molecules, imaging probes, photosensitisers, etc. (Kim et al., 2020). A unique property of dendrimers is their chemical and physical stability inherent to their chemical structure (Kalomiraki et al., 2016).

Dendrimers are extensively branched synthetic macromolecules having a well-defined structure and composition. These molecules are produced by the repeated assembly of polymer layers over the core. There are many dendrimer types, including peptide, poly(L-lysine), polyamideamine (PAMAM), silicone, polyethyleneimine, and other dendrimers. PAMAM dendrimers are the most extensively studied ones as drug and gene delivery systems. They form stable complexes with DNAs, siRNAs, and miRNAs referred to as dendriplexes. These complexes show high transfection efficacy and ability to protect nucleic acids from damage (Fant et al., 2008). Their modifications make it possible to create the derivatives possessing reduced toxicity, increased gene delivery specificity and efficacy (Abedi-Gaballu et al., 2018; Liu C. et al., 2019).

In the recent decade, three common strategies for dendrimer modification are used: (1) surface modification with different moieties (Yang J. et al., 2015); (2) hybrid vector formation (Biswas et al., 2013); (3) creation of self-assembling supramolecular nanoparticles (Yadav et al., 2020).

The first strategy is exemplified by the studies by Nagasaki T. et al. summarized in a review (Nagasaki, Shinkai, 2007), which used a cationic polyazobenzene dendrimer modified with L-lysine (Lys-G2). The dendrimer complex with a plasmid DNA ensured increased transfection efficacy when administered to cytoplasm and UV-irradiated

The second strategy implies conjugation of ligands, polymers, inorganic nanoparticles, etc. with the dendrimer complex surface (Lin et al., 2018), which improves the dendrimer carrier properties. Mbatha L.S. et al. (Mbatha et al., 2021) have developed hybrid carriers by means of derivatization of gold nanoparticles with folic acid and 5th generation polyamidoamines. Their cytotoxicity and transgene expression efficacy were assessed in vitro using a luciferase reporter gene. The hybrid vectors ensured an increased luciferase expression vs. PAMAM dendrimers with folic acid or unbound dendrimers

An example of the third strategy is a study aimed at building supramolecular nanoparticles of variable size (30–450 nm) from three different units, PAMAM dendrimer with adamantane, branched polyethyleneimine conjugated with cyclodextrin, and polyethylene glycol with adamantane (Lu S. et al., 2020). These nanoparticles were used as a vector for the anti-cancer RNA interference agents, which resulted in reduced vascularization and inhibition of the lung tumor xenograft growth in a mouse model

A study (Zarebkohan et al., 2015) yielded a PAMAM-PEGdendrimer coated by serine-arginine-leucine (SRL) tripeptide for the delivery of genes into C6 glioma line cells. The results showed that such nanoparticles efficiently transfected the brain tumor cells.

Thus, dendrimers are a promising means for the delivery of genetic materials and genome editing tools. However, one of their critical limitations is related to their toxicity, with 3th to 5th generation dendrimers being less toxic than higher generations (Shcharbin et al., 2013). Moreover, dendrimer cytotoxicity depends on their branch elasticity (Tang et al., 1996), hydrophobic properties, the number and nature of the surface and core modifications (Somani et al., 2018). A broad range of modifications changing these parameters allows selection of the most suitable ones in order to minimize adverse effects of dendrimer-based vectors

## Polymer nanoparticles

Polymer nanoparticles possess chemical variety and have great potential due to their flexible structural modifications. They are widely used to deliver nucleic acids and other substances into cells and tissues

These carriers are built from various natural and synthetic polymers. Natural macromolecules have a number of advantages over synthetic ones, which are generally consigned to the lack of toxicity, relatively low cost and ease of preparation. They include celluloses, starches, gelatin, collagen, chitosan, agar, pectin, inulin, dextrin, etc. These biopolymers can be modified to create delivery systems addressing particular tasks (Yadav et al., 2020; Basinska et al., 2021). For example, chitosan is the natural polymer that is most commonly used for CRISPR/Cas9 delivery. Its main advantages are biocompatibility, biodegradability, and lack of cytotoxicity. Qiao J. et al. encapsulated red fluorescent protein and Cas9/ribonucleoprotein fused to a polyglutamate peptide tag together with donor DNA into the chitosan nanoparticles. The polymer carrier ensured simultaneous delivery of both the genome editing tool and the single-strand DNA matrix while showing highly efficient transfection of HeLa cells with no cytotoxicity (Qiao et al., 2019).

The list of synthetic polymers is also large enough. Among them, the most explored delivery means include polylactic and polyglycolic acids, their copolymers, polycaprolactam, polyhydroxybutyrate, etc. They possess good biocompatibility and biodegradability, which support their wide use in medicine, biotechnology, agriculture and other fields (Singh A.V., 2011; Zhang S. et al., 2021).

There are reports of the ongoing development of complex carriers comprising several polymers at once, which allows to overcome the drawbacks of particular components owing to the advantages of others. Thus, in (Luo et al., 2018), a block copolymer of polyethylene glycol, β-poly(lactic-glycolic) acid and cationic lipids was used to obtain specific nanoparticles for the delivery of Cas9 mRNA and CRISPR/Cas9 plasmids into the macrophages. The resulting carriers induced specific Cas9 expression in the macrophages and monocytes both in vitro and in vivo

Rui Y. et al. synthesized polymers from carboxylated branched poly(β-amino esters) by stepwise copolymerization. Their results showed that C5-caped polymer ensured maximum cargo release efficacy after absorption by the cells. Furthermore, it was used to produce the nanoparticles for CRISPR-Cas9 ribonucleoprotein encapsulation. The authors found that the delivery of genome editing tools led to 77 % and 47 % knockout of the target gene in HEK-293T and GL261 mouse glioma cells, respectively (Rui et al., 2019).

Therefore, polymeric nanoparticles are generally safe, easy to produce and customizable. Moreover, they undergo degradation in the host body and are suitable for all strategies of CRISPR-Cas9 delivery. However, the efficacy of delivery using the polymeric carriers is thought to be insufficient (Liu C. et al., 2019).

## Gold nanoparticles

A number of studies propose gold nanoparticles as a vector base to address the issues with in vitro and in vivo delivery of genome editing tools. It was shown that small (<3 nm) gold nanoparticles are biocompatible but possess cytotoxicity and immunogenic potential (Shukla et al., 2005). Gold nanoparticles can be bound with various ligands, drug molecules, genome editing tools, which expands their applications

Gold nanoparticles used to transfer ribonucleoproteins for genome editing into the brain cells showed no cytotoxicity or adverse effects on the neuron function (Lee et al., 2018). A paper (Glass et al., 2017) describes efficient elimination of a DNA mutation leading to Duchene muscular dystrophy in a mouse model using gold nanoparticles carrying CRISPR components, with minimum off-target effects. In another study (Jia et al., 2017), gold nanoparticles covalently conjugated with a siRNA successfully delivered their cargo into the macrophages, which resulted in the inhibition of inflammation and restoration of the heart function in a laboratory animal cardiomyopathy model.

In a study (Mout et al., 2017b), arginine-coated gold nanoparticles were conjugated with the synthetic constructs of ribonucleoproteins and Cas9 oligoglutamate-tagged protein. These complexes were incubated with HeLa, HEK-293T, and Raw 264.7 cell cultures. The delivery system ensured highly efficient (about 90 %) transfer of Cas9 and ribonucleoproteins into the cytoplasm and nucleus, with 23 to 30 % genome editing efficacy. Tao Y. et al. (Tao et al., 2021) have shown the suitability of surface-modified gold nanoparticles for real-time monitoring of the biological effects during genome editing.

The limitations of gold nanoparticles include a lack of knowledge on the correlation of their immunogenic potential and toxicity with appropriate physicochemical properties, such as size, shape, charge, and surface modifications (Dykman, Khlebtsov, 2017). The approaches to reduce toxicity of such carriers and improve the delivery efficacy include the use of the complex nanoparticles comprising polyethyleneimine, polyethylene glycol, and other components promoting reduction in the immunogenic properties of the particles and preventing their binding to off-target receptors (Li Y. et al., 2017).

## Metal-organic frameworks

Current studies in the field of abiotic vectors include extensive development of the carriers derived from metal-organic frameworks (MOFs) as the non-viral vehicles to deliver nucleic acids into target cells. MOFs are a novel class of porous materials. Their crystal lattice is formed by coordinate bonds between the central alkaline-earth or transition metal ions (Ca, Mg, Zn, Ti, Zr, Mn, Pd, Cu, Cr, Cd, etc.) and organic ligands having chelating moieties (Cheetham et al., 1999; Valtchev et al., 2009; Farha et al., 2012; Paz et al., 2012; Furukawa et al., 2013; Yu Y. et al., 2013; Li H. et al., 2018; Corella-Ochoa et al., 2019). MOF synthesis produces high-ordered porous crystal structures with strictly defined pore parameters (Wang Z., Cohen, 2009). Moreover, the MOF technology allows controlling the porosity and pore size in accordance with the cargo properties.

In addition, particular MOFs (e. g., based on zinc, calcium, magnesium, titan, zirconium, iron ions and biocompatible organic ligands, including polycarbonates, imidazolates, amines, phosphates, etc.) are biodegradable and low-toxic (Horcajada et al., 2012; Lyu et al., 2021). Therefore, such MOFs are widely used in experimental medicine as controlled-release drug carriers (Su et al., 2015; Ranjbar et al., 2018; Chen G. et al., 2019; Osorio-Toribio et al., 2020). A nanosized zeolitelike framework based on imidazole and zinc salts (ZIF) is particularly useful for these purposes. It has low toxicity, broad controllability of pore parameters, buffer properties and endosomal escape ability (Alsaiari et al., 2018). In a number of studies, MOFs are used for encapsulation of biologically active compounds such as insulin (Chen Y. et al., 2018), heparin (Vinogradov et al., 2018), hemoglobin (Peng et al., 2019). Moreover, a research team (Liang et al., 2016) successfully encapsulated living cells into a MOF, which ensured their preservation and physical protection

Encapsulation of genome editing tools in the pores of such materials prevents their degradation in the physiological conditions until they reach their targets (Peng et al., 2018). There are two mechanisms of encapsulation of genome editing tools into the MOFs. The first one is the encapsulation by direct absorption into the pores. For example, a paper (Teplensky et al., 2019) describes the encapsulation of an RNA molecule into the pores of NU-1000, a zirconium-based MOF. The second mechanism is the biomineralization, i. e. the building of a metal-organic framework over the material to be encapsulated (Li Y. et al., 2019).

In a study (Alsaiari et al., 2018), the encapsulation of CRISPR/Cas9 into ZIF-8 was described. The cargo weight reached 1.2 % of the total polymer weight, with 17 % pore loading efficacy, which the authors considered a good result in contrast to previously reported values for the MOF-based delivery systems. The polymer showed no cytotoxic properties in concentrations up to 200 mg/mL, was stable in physiological conditions but was rapidly destroyed at pH of 5–6, which creates the potential for controlled cargo release in vivo. This complex also had an enhanced endosomal escape ability over the cationic lipid-based vehicles and reduced target gene expression twofold when incubated for 2 days and threefold when incubated for 4 days, which was two times higher than the efficacy of the target gene knockdown with lipofectaminemediated CRISPR/Cas9 delivery.

Specific delivery to the target cells is critical for improving the genome editing efficacy and safety. Alyami M.Z. et al. proposed a coating for ZIF-8 with encapsulated CRISPR/Cas9, which was based on MCF-7 human breast adenocarcinoma cell membrane. Incubation of such modified MOF with MCF-7, HeLa, HDFn, and aTC cells showed that MCF-7 possessed the maximum carrier absorption efficacy while the other cell lines absorbed the agent to a small extent. Moreover, such a composite, when transfected to the MCF-7 cells, inhibited EGFP expression threefold vs. the HeLa membrane-coated ZIF (Alyami et al., 2020

Currently, there is an ongoing discussion of particular chemistries useful for the controlled delivery of Cas9/gRNA into the cells using MOFs in the presence of endogenic or external signals (Yang X. et al., 2019; Lyu et al., 2021). Thus, the carrier systems that penetrate the cells by endocytosis come to the organelles with an acid content, such as endosomes or lysosomes. Considering intracellular pH levels, the pHsensitive hybrid carriers were created from silicon dioxide and ZIF (SMOFs) for efficient encapsulation and delivery of hydrophilic compounds (Wang Y. et al., 2020). SMOF nanopaticles with encapsulated ribonucleoproteins ensured efficient genome editing in vivo in the mouse retinal pigment epithelium after subretinal injection.

In addition, abnormal cells and tissues often have a unique microenvironment with specific levels of pH and other active substances such as enzymes and ATP which could be used for MOF-mediated targeted delivery. Yang X. et al., relying on the activation of ATP production in some disorders, created an ATP-sensitive zeolite-like framework based on imidazole and zinc ions (ZIF-90). This material efficiently encapsulated CRISPR/Cas9 and ensured delivery of a large amount of protein payload into the cell matrix, regardless of the particle size and molecular weight. In the presence of ATP, ZIF-90/protein conjugates were destroyed, releasing the protein due to competitive coordination between ATP and Zn2+ in ZIF-90. After transfection, target gene expression in HeLa cells was inhibited by up to 35 % (Yang X. et al., 2019)

The study by Chen T.T. et al. also showed that ZIF-8 nanoparticles were able to release encapsulated proteins rapidly in acid media but not at рН 7.4 (Chen T.T. et al., 2018), which may be preferred in some disorders

Despite certain advances and potential of MOFs as vectors for genome editing tools, there are also issues yet to be addressed. Particularly, there is a need for the following studies: (1) to improve the specificity and efficacy of targeted effects of MOF nanoparticles; (2) to increase MOF/biomolecule conjugate stability in the bloodstream with intravenous administration; (3) to find ways for reducing the immunogenic potential and toxicity of MOFs; (4) to estimate the long-term safety of the carriers; (5) to finalize the large-scale production of the carriers with defined parameters (Lyu et al., 2021; Zheng et al., 2021).

## Conclusion

Currently, there is a variety of methods and systems for the delivery of genome editing tools. They have both unique advantages and drawbacks. At the same time, it is worth acknowledging that a single universal carrier for delivery of all types of the agents cannot be developed. It seems clear that the choice and use of certain viral or non-viral vectors must primarily be defined by the specific aspects of the problem to be solved. In particular, synthetic carriers are preferred for the simultaneous loading of several substances and components, which is especially relevant for genome editing. Therefore, there is an increasing number of proposals to combine different non-viral delivery systems. For example, in a number of cases, it makes sense to deliver the genes and therapies using cellpenetrating peptides combined with nanoparticles, micelles, liposomes, or polymers. In this context, MOF-based carriers, which allow the implementation of a broad spectrum of capabilities, have great potential. Further development of such vectors and technologies can result in safe and efficient delivery systems that would be able to circulate in the body for a long time while recognizing target cells and ensuring cell-specific delivery and release of intact cargoes.

## Conflict of interest

The authors declare no conflict of interest.
